# Case of Extraction of the Bones of a Fœtus

**Published:** 1858-08

**Authors:** D. W. Young

**Affiliations:** Aurora, Ill.


ARTICLE II.

### REPORT OF A CASE OF EXTRACTION OF THE BONES OF A FŒTUS FROM THE PERITONEAL CAVITY, AFTER HAVING BEEN THERE OVER FOUR YEARS.

BY D. W. YOUNG, M. D., AURORA, ILL.

On the 17th day of August, 1855, I was summoned to visit Mrs. Phillip Youngels, a large and robust German lady, residing one and a half miles north of this city in what is called the *Big Woods*, whom I found in an advanced stage of labor with her second child.

She was already far advanced in labor when I arrived, and consequently required but few more pains to complete it.

The child, a large, well formed male, weighed some eight or nine pounds, and was strong and healthy.

After the child was born and the placenta removed, and while I was applying the bandage, she directed my attention to a tumor in her right side, situated in the iliac region, which she informed me had been there for something over four years, and gave the following interesting history of its commencement.

She says that while she was still residing in Germany, she became, as she supposed, pregnant for the first time; that she enjoyed pretty good health during the first six months of gestation, but during the latter three or four months she suffered quite a good deal of pain in her right side.

At or about the expected time for her confinement, pains came on, and she and her friends supposed that labor had really commenced, and accordingly called their midwife, who came, and after repeated examinations expressed her fears that the child did not *lay right*, as she could not reach it, and as it did not advance properly notwithstanding the severe pains. Her pains continued and increased until she says something burst and discharged a large quantity of water and bloody matter, together with clots of coagulated blood, after which the pains ceased; the tumor diminished in size and moved higher up, and farther to the right side. The final conclusion with them was that she had only suffered from obstructed menstruation which had now been relieved. The lochial discharge continued for two or three weeks, during which time the tumor also diminished somewhat in size. But finally the lochial discharge ceased, leaving the present tumor, which to all appearance has remained perfectly dormant up to this time.

A few months subsequently she consulted her surgeon in relation to the tumor, who informed her that the tumor was most undoubtedly an ovarian tumor which could only be relieved by a very hazardous operation, which, while she suffered so little inconvenience, he would not advise.

She soon again became pregnant and in due time was delivered of a living healthy child. Parturition was natural and easy, and she soon regained her usual degree of health, having experienced nothing different from other parturient women under the circumstances, except an entire absence of the lacteal secretion. The tumor apparently remaining in the same dormant condition.

Some two years subsequently she again became pregnant, and in August, 1855, I attended at her confinement, which, as I have above stated, was uncomplicated and easy.

As the tumor remained, to all appearance, just as it had been for over four years, of course I advised no interference unless necessity should require it.

I heard nothing more from Mrs. Youngels until the first of May, 1856, when her husband called at my office to consult me in relation to his wife, who he said was very poorly. He informed me that some time after her confinement she was attacked with chills and fever, and that as his friend, A. W. Heise, M. D., a very eminent German physician and surgeon from Addison, Du Page Co., Illinois, was in the neighborhood, he called him to prescribe for his wife. Notwithstanding the medicine, the chills and fever continued; the tumor became painful, inflamed, and finally opened and discharged several small bones, one of which he exhibited, and I pronounced it the fibula of the right side. He said that he had that day written to Dr. Heise, requesting him to visit his wife immediately, and desired that I would see her with him when he came. I promised, and he returned home.

On the 5th day of May, 1856, I was summoned to meet Dr. Heise in consultation, who was then waiting at her house.

At my request, P. A. Allaire, M. D., of this city, was also called to examine the case in consultation.

The patient was very much emaciated, and seemed suffering from hectic fever and severe night sweats, together with extreme nervous prostration. The tumor near the umbilicus was ulcerated and discharging a *most terribly* offensive matter. After due consultation, and at the earnest solicitations and request of the patient, an operation was decided upon, notwithstanding her extreme emaciation and feebleness.

Accordingly, I put the patient thoroughly under the influence of chloroform, when the operator, Dr. Heise, proceeded with a grooved director and bistoury to make an incision of some inch or two toward the right side, and with a long pair of forceps commenced to extract the bones. The bones of the head and pelvis were too large to pass through the small orifice, which seemed as large as we dare make for fear of cutting beyond the adhesions of the sac and peritoneum, consequently they were broken up with a strong pair of scissors. This was a very slow and tedious job, as you will notice that the bones are perfect and fully of the size of an ordinary nine months' fœtus, and very firmly ossified, being as hard or even harder than the bones of an adult skeleton. Drs. Heise and Allaire took turns in operating, and were full two hours in removing all the bones and cleansing the sac. The patient was during all this time kept thoroughly under the influence of chloroform with the most happy effect. In fact, when the operation was completed, and she permitted to come from under its influence, she expressed herself as feeling decidedly better.

They succeeded in removing all the bones, together with a large mass of semi-decomposed matter, which was most indescribably offensive, from the sac. The sac itself was firm, dense, and so tough as almost to resist the scalpel, and was contracted closely around the bones. After the operation, the dressing consisted of a simple broad bandage around the abdomen, with directions that she be kept on her side so as to facilitate the discharge from the wound.

I visited her the next morning as per agreement, and found her comfortable and in the position we advised when we left her. She was a little hoarse and complained some of her throat, doubtless from the effect of the chloroform. I removed the bandage, when the wound discharged quite profusely; reapplied the bandage and continued the same directions.

Things progressed thus favorably for four or five days, when I discovered that all or nearly all the fæcal matter was being discharged from the wound and through the orifice of the sac, near the umbilicus. I invited Drs. Howell and Allaire to visit her with me. They both examined her thoroughly, and found as I had that the fæces were really passing through the wound.

The plan of treatment instituted for this formidable complication was as follows:

I applied one large compress above and another below and close against the walls of the sac, then one immediately over the orifice, and then a broad bandage firmly applied over them around the abdomen, with directions that her bowels must *positively* be moved once a day either by castor oil or injections. I also gave her freely of good nutritious food with tonics, forbidding them to remove the bandage or compresses in my absence.

Under this plan of treatment, I soon had the satisfaction of seeing our patient improve very rapidly. The bowels soon moved regularly *by the rectum*, the discharge from the sac decreased, the sac contracted, adhesions formed, the orifice closed, and at the end of four months I discharged her perfectly cured from *all her pregnancies*, *ulcerated bowels*, *artificial anus*, *and all difficulties of whatsoever kind*, and up to this time, two years and two months, she has enjoyed excellent health.

[The bones referred to by Dr. Young, upon examination, proved to be: eleven ribs, two ulnæ, one radius, two humeri, two femurs, two tibia, two fibula, one scapula, one clavicle, two ilia, sphenoid, various and numerous fragments of bones of the head, etc., etc. Not a trace of the vertebra or bones of the hands or feet, among the lot sent, can be found. The bones are in a fair state of preservation, and quite as large as we might expect those of a full grown fœtus to be. There are several interesting points in this case to which I might refer, but I will only take the liberty now of mentioning one, that is the character of the pregnancy. I think there is quite sufficient reason to doubt whether it was a case of extra-uterine fœtation proper. So far as the history is given (which I own is too imperfect upon which to base positive conclusions), it favors the supposition of rupture of the uterus, extrusion and peritoneal imprisonment of the fœtus. I need but mention that the discharge of a large quantity of water, clots of blood and bloody matter, and the removal of the tumor farther up in the abdomen, are unlikely if not impossible phenomena in the spurious labor of extra-uterine pregnancy. It is almost as difficult, however, to account for the escape of the patient from fatal peritonitis under this supposition, as for the unusual circumstances above mentioned occurring in real extra-uterine fœtation. W. H. B.]

---

ARTICLE III.

